# Longitudinal Survival Outcomes in Allogeneic Stem Cell Transplantation: An Institutional Experience

**DOI:** 10.3390/cancers14225587

**Published:** 2022-11-14

**Authors:** Justin Jiang, Audrey M. Sigmund, Qiuhong Zhao, Patrick Elder, Don M. Benson, Sumithira Vasu, Samantha Jaglowski, Alice Mims, Hannah Choe, Karilyn Larkin, Jonathan E. Brammer, Sarah Wall, Nicole Grieselhuber, Ayman Saad, Sam Penza, Yvonne A. Efebera, Nidhi Sharma

**Affiliations:** 1 College of Medicine, The Ohio State University, Columbus, OH 43210, USA; 2 Department of Internal Medicine, Division of Hematology, The Ohio State University, Columbus, OH 43210, USA; 3 Division of Hematology, Blood and Marrow Transplant, OhioHealth, Columbus, OH 43214, USA

**Keywords:** allogenic transplantation, overall survival, progression-free survival, graft-versus-host disease

## Abstract

**Simple Summary:**

Stem cell transplantation from a donor can be a potential cure for several blood cancers. We have maintained the characteristics and outcomes of patients receiving these transplants at our institution, the Ohio State University, since 1984. In this study, we looked at these outcomes, including how long patients lived after transplant, how long before their cancer came back, and if they developed other complications related to receiving a transplant, and evaluated how they changed over the years. We conclude that patients are now living longer with a lower chance of their cancer returning or of developing other complications, likely as a result of improvements in supportive care for transplant patients.

**Abstract:**

Allogeneic hematopoietic stem cell transplantation (allo-SCT) is a potentially curative treatment for many hematological disorders, but is often complicated by relapse of the underlying disease, graft-versus-host disease (GVHD), and infectious complications. We conducted a retrospective analysis on patients undergoing allo-SCT from 1984 to 2018 to better understand how survival has changed longitudinally with therapeutic advancements made to mitigate these complications. Method: We analyzed data from 1943 consecutive patients who received allo-SCT. Patients were divided into groups (gps) based on the year (yr) of transplant. Primary endpoints were overall survival (OS), progression free survival (PFS), and GVHD-free relapse-free survival (GRFS). Secondary endpoints were the cumulative incidences of grade II–IV and grade III–IV acute GVHD (aGVHD), chronic GVHD (cGVHD), and non-relapse mortality (NRM). Results: Our study found statistically significant improvements in OS, PFS, and GRFS. Five-year PFS among the groups increased from 24% to 48% over the years. Five-year OS increased from 25% to 53%. Five-year GRFS significantly increased from 6% to 14%, but remained relatively unchanged from 2004 to 2018. Cumulative incidences of grade II–IV aGVHD increased since 2009 (*p* < 0.001). However, cumulative incidence of NRM decreased since 2004 (*p* < 0.001). Conclusions: Our data show improved OS, PFS, and GRFS post allo-SCT over decades. This may be attributed to advances in supportive care and treatments focused on mitigation of GVHD and relapse.

## 1. Introduction

In the past 50 years, over one million hematopoietic stem cell transplants have been performed, with approximately 42% percent being allogeneic hematopoietic stem cell transplants (allo-SCTs) [1]. While allo-HCT provides the potential for cure for patients with hematologic disease who may not otherwise have one, it is not without significant toxicity and challenges. The cornerstone of allo-SCT is the potential for the graft-versus-leukemia (GVL) or graft-versus-disease effects, which harness donor immunity to improves disease therapy outcomes [2,3,4]. Where conventional chemotherapy targets actively proliferating malignant cells, GVL offers the additional benefit of targeting quiescent malignant cells potentially responsible for relapse, making allo-SCT valuable in hematologic malignancies with poor prognostic factors (e.g., cytogenetics) or with relapse/refractory disease. However, GVL also comes with the risk of graft-versus-host disease (GVHD), which can result in significant morbidity and mortality for patients. In the early era of allo-SCT, pre-transplant conditioning that involved myeloablative (MA) regimens with high-dose total body irradiation theoretically suppressed the immune system of the transplant recipient [5]. Allo-SCT then expanded to use reduced-intensity conditioning (RIC) regimens in an effort to reduce organ toxicity, non-infectious pulmonary complications, and other causes of non-relapse mortality (NRM) [6,7]. However, GVHD persists as an obstacle following allo-SCT. Many strategies have been used to reduce the risk of acute GVHD, including the use of calcineurin inhibitors such as cyclosporine and tacrolimus, antithymocyte globulin (ATG), post-transplant cyclophosphamide, and other novel agents [8,9,10,11]. Beyond GVHD, allo-SCT patients universally experience extensive immunodeficiency, leaving recipients susceptible to a host of infections. These infections include cytomegalovirus (CMV), Epstein–Barr virus (EBV), and other respiratory viruses, each of which have different prophylaxis and treatment strategies. The implementation of these strategies has taken place in the past few decades with limited longitudinal evidence of their significance.

Multiple single institution and registry database studies have evaluated time–trend analysis of transplant-related outcomes over time [12,13,14,15,16,17]. Although these studies varied from single institutional studies to large European Society of Bone and Marrow Transplant (EBMT) database, our study adds to the consensus of sustained improvement in survival outcomes in the past two decades compared with the 1980s through the 1990s. With the expansion of donor registries in recent years as well as advancements in haploidentical and alternate donor transplant protocols, nearly all patients indicated for allo-SCT can gain access to a donor in the United States [18]. At the same time, more treatment options have emerged with the development of novel therapies, including targeted immunotherapies, small molecule drugs, and chimeric antigen receptor T-cell therapies, providing a wide range of treatment options. Therefore, these data are important in deciding the future role and directions of allo-SCT in hematological conditions in light of these other therapies.

Hence, through this study, we investigated survival outcomes and GVHD incidence across the years from 1984 to 2018 at the Ohio State University.

## 2. Methods

### 2.1. Patients and Study Design

This study included all adult patients (age > 18) who received their first allo-SCT at the Ohio State University from 1984 to 2018. Patients under the age of 18 were excluded. Patients were divided into seven groups based on the year of transplant consisting of 5-year increments: 1984–1988, 1989–1993, 1994–1998, 1999–2003, 2004–2008, 2009–2013, and 2014–2018. These increments were selected to maximize the relative distribution of patients as well align roughly with institutional changes in the transplant protocol.

Data collected in this study included demographic information including age, self-reported race, gender, and zip code, as well as disease type, treatment regimens and responses, Karnofsky Performance Scale (KPS), and comorbidity index (CI). The following transplant characteristics were also collected: donor type (match, mismatch, related, unrelated), tissue type (bone marrow, peripheral blood, or cord), graft-versus-host-disease (GVHD) prophylaxis, CD34 amount infused, day of neutrophil and platelet engraftment, human leukocyte antigen (HLA) loci match, gender of receipt, gender of donor, and CMV and EBV status of both the recipient and donor. Neutrophil engraftment was defined as the first of 3 consecutive days with an ANC ≥ 0.5 × 10^9^/L. Platelet engraftment was defined as the first of 7 days with a platelet of ≥20 × 10^9^/L without platelet transfusion. Conditioning regimen and type were included as defined by the Center for International Blood and Marrow Transplantation (CIBMTR) research definitions for myeloablative (MA) and RIC [19]. Transplant-related complications like infections, veno-occlusive disease (VOD), hemorrhagic cystitis, and acute and chronic GVHD were collected. The dates of last follow up and relapse were also collected. This study was approved by the clinical scientific review committee and institutional review board at the Ohio State University.

### 2.2. Endpoints

The primary objective of this study was to assess how survival changed at our institution from 1984 to 2018. The primary endpoints of overall survival (OS), progression free survival (PFS), and GVHD-free relapse-free survival (GRFS) were used to compare outcomes across transplant years. The composite endpoint GRFS was assessed as the absence of grade III–IV acute GVHD (aGVHD), chronic GVHD (cGVHD) requiring system treatment, relapse, or death [20]. The secondary endpoints were the cumulative incidences of grade II–IV and grade III–IV acute GVHD (aGVHD), chronic GVHD (cGVHD), and non-relapse mortality (NRM). Classification of aGVHD was based on the Glucksberg grade, while cGVHD was classified using standard definitions [21,22].

### 2.3. Statistical Analysis

The Mann–Whitney U test for continuous variables and chi-squared or Fisher’s exact test for categorical variables were used to compare patient-, disease-, and transplant-related characteristics among the seven groups. The Kaplan–Meier method was used to calculate the probabilities of OS, PFS, and GRFS and they were compared using the log-rank test. Owing to the differences in length of follow-up, considering the median follow-up of 3.5 years in the most recent transplant group, all of the survival outcomes were censored at 3 years in the analysis. Gray’s test, accounting for competing risks, was utilized to estimate cumulative incidence rates for relapse, NRM, and acute and chronic GVHD. The competing risks for GVHD were relapse and death; the competing risk for relapse was death; and the competing risk for NRM was disease-related deaths. A *p*-value of <0.05 was considered statistically significant. Analyses were performed using Stata 16 (College Station, TX, USA).

## 3. Results

### 3.1. Patient and Disease Characteristics

Our analysis included 1943 consecutive patients who received an allo-SCT at the Ohio State University from 1984 to 2018 (Table 1). The median age at time of transplant was 50.0 years (range: 18–76), with 59.6% of the patients being male (n = 1158) and 94.3% being Caucasian (n = 1830). Acute myeloid leukemia (AML) accounted for the majority of transplants, at 36.3% of transplants, followed by non-Hodgkin lymphoma (14.2%), acute lymphoid leukemia (11.8%), chronic myeloid leukemia (10.1%), and myelodysplastic syndrome (10.0%). Across the time periods, the distribution of gender and race was similar across the groups. However, we did observe a significant increase in age across time with median age starting at 31.0 years in 1984–1988 to 57.0 years in 2014–2018 (*p* < 0.001). Complete comorbidity index data were available from 2004 based on the hematopoietic stem cell transplant comorbidity scoring system (HCT-CI) developed by Sorror et al [23]. Most patients had a comorbidity index of 0–1 and 2–3 in 2004–2008, while 2009–2013 and 2014–2018 had a larger patient population with HCT-CI scores of 4+.

### 3.2. Transplant Characteristics

As expected, the allo-SCT performed across the years consisted mainly of match-related and unrelated donors with 46.7% (n = 908) and 38.9% (n = 756), respectively (Table 1 and Table 2). Yet, with the expansion of donor registries, we observed a larger ratio of match-unrelated donors compared with match-related, starting with 66.5% match-related (n = 147) and 25.8% match-unrelated (n = 57) in 1999–2003 to 28.0% match-related (n = 168) and 53.8% match-unrelated (n = 322) in 2014–2018. Additionally, starting in 2009–2013 and continuing into 2014–2018, we started to see increased utility of haploidentical transplant. In terms of conditioning regimen, 55% of patients received myeloablative conditioning (n = 1068), while 45% received reduced-intensity conditioning (n = 875). In recent years (2014–2018), there has been increased utility of reduced intensity conditioning, with 45.2% receiving myeloablative conditioning (n = 271) and 54.8% receiving reduced-intensity conditioning (n = 328). For GVHD prophylaxis, most patients received tacrolimus-based GVHD prophylaxis (82.3%, n = 1249), while another 15.8% received cyclosporin-based GVHD prophylaxis (15.8%, n = 240), which reflects our institutional preference.

### 3.3. Acute Graft-versus-Host Disease and Non-Relapse Mortality

**Figure 1:**
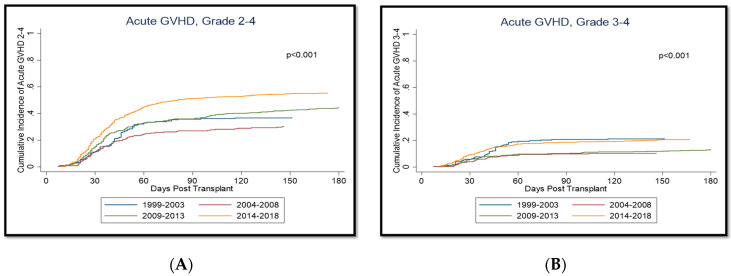
Cumulative incidences of aGVHD. (**A**): aGVHD, Grade II-IV; (**B**): aGVHD, Grade III-IV.

Complete GVHD data were available starting with the 1999–2003 group. The cumulative incidence of grade II–IV aGVHD increased over the years with 36%, 27%, 38%, and 52% at day 100 and 37%, 31%, 44%, and 55% at day 180 from the 1999–2003 to the 2014–2018 group (Figure 1, Table 3), while the corresponding incidence of grade III–IV aGVHD was 21%, 10%, 11%, and 19% at day 100 and 22%, 11%, 13%, and 21% by day 180 from the 1999–2003 to the 2014–2018 group. The highest rates of grade III–IV aGVHD were seen in the 1999–2003 and 2014–2018 groups, with no significant difference between these groups. Subsequently, Cox proportional hazard models for both grade II–IV and III–IV aGVHD were constructed, adjusting for age at allo-SCT, receipt-donor gender, race, zip code, diagnosis, donor type, conditioning, and remission status at transplant. These models confirmed our previous findings with a significant increase from 1999–2003 to 2014–2018 for grade II–IV aGVHD (*p* < 0.001) and no significant difference for grade III–IV aGVHD (*p* > 0.655) over the years. In our grade II–IV aGVHD model, a significantly increased risk for grade II–IV aGVHD in patients was seen with matched unrelated, haploidentical, and mismatch-unrelated donors compared with match-related donors (*p* < 0.001). However, a significantly increased risk for grade III–IV was only seen in patient with mismatched-unrelated donors (*p* = 0.048). Both grade II–IV and III–IV aGVHD risk was also significantly increased in patients receiving myeloablative conditioning compared with those receiving reduced-intensity conditioning (*p* < 0.001 and *p* = 0.047, respectively). The incidence of cGVHD (both extensive and limited) increased - across the years.

**Figure 2:**
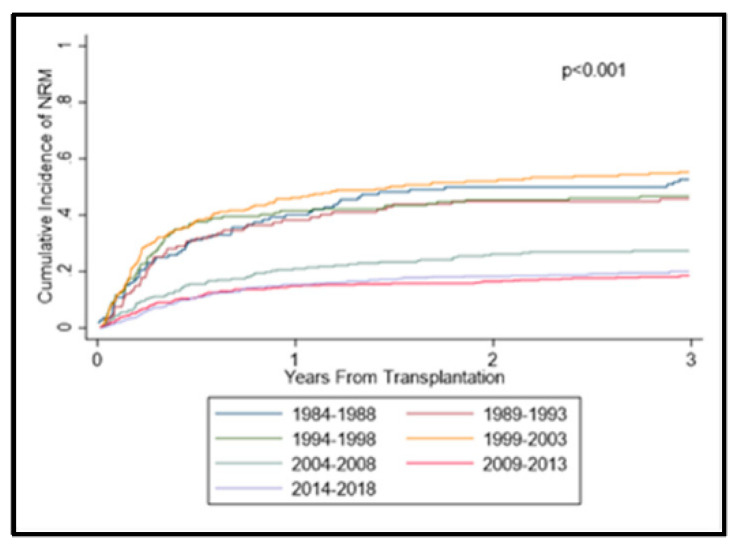
Cumulative incidences of non-relapse mortality.

The cumulative incidence of NRM significantly improved across the years, with 1-year NRM at 40%, 38%, 42%, 46%, 21%, 15%, and 15% and 5-year NRM at 54%, 51%, 51%, 57%, 31%, 22%, and 24% from 1984–1988 to 2014–2018 (Figure 2). A Cox proportional hazard modeling for NRM was also constructed with the same parameters as the previous aGVHD model (data not shown). The modeling suggested a significant improvement in NRM beginning with 2004–2008 and continuing to 2014–2018 (*p* < 0.001) compared with 1984–1988.

### 3.4. Relapse

**Figure 3:**
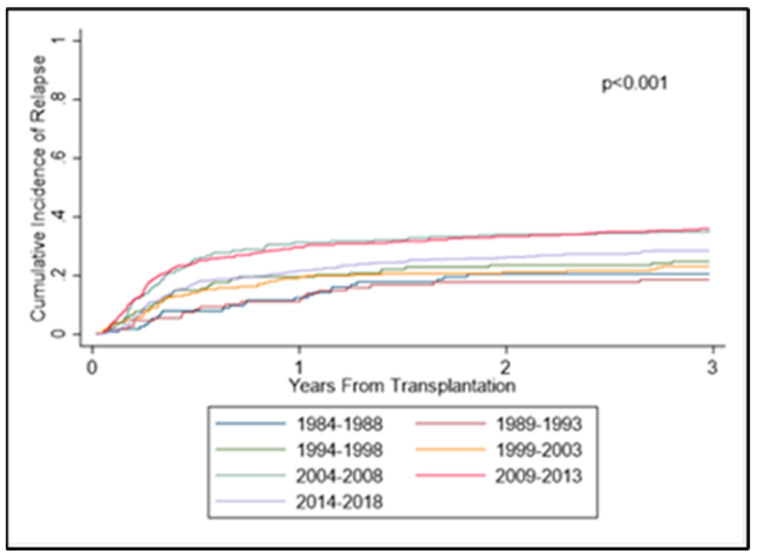
Cumulative incidences of relapse.

The cumulative incidence of relapse increased across the years at both the 5-year and 10-year marks. The rate of 5-year relapse was as follows: 22% (1984–1988), 21% (1989–1993), 28% (1994–1998), 24% (1999–2003), 38% (2004–2008), 38% (2009–2013), and 29% (2014–2018). The rate of 10-year relapse was as follows: 23% (1984–1988), 24% (1989–1993), 28% (1994–1998), 25% (1999–2003), 40% (2004–2008), 39% (2009–2013), and not reached (NR) (2014–2018). After adjusting for the covariates as described previously, similar results were found with significantly increased relapse in the last three groups (Figure 3). Notably, matched-unrelated/mismatch-unrelated showed significantly decreased risk for relapse compared with match-related donors (*p* = 0.004 and *p* = 0.019, respectively). Reduced-intensity conditioning predicted significantly higher risk for relapse compared with myeloablative conditioning ((*p* < 0.001); Figure 3, Table 4).

### 3.5. Survival Trends across Time Periods

**Figure 4:**
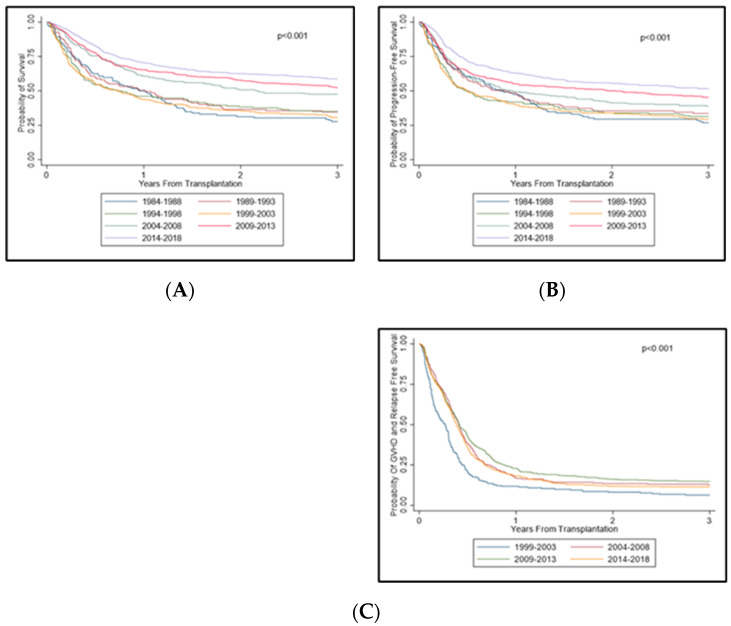
Kaplan-Meier curves. (**A**): OS; (**B**): PFS; (**C**): GRFS.

The median follow-up time was 31.3, 26.7, 22.2, 17.2, 12.9, 8.1, and 3.5 years, respectively, across the years 1984–1988, 1989–1993, 1994–1998, 1999–2003, 2004–2008, 2009–2013, and 2014–2018. From 1999 to 2018, Kaplan–Meier analysis showed statistically significant improvements in PFS, OS, and GRFS (*p* < 0.001 for all; Figure 4). Median PFS improved from 0.8 years (95% confidence interval [CI]: 0.6–1.2) in 1984–1988 to 3.7 years (95% CI: 2.3-NR) in 2014–2018, with an overall median PFS of 1.3 years (95% CI: 1.1–1.5). The median OS also improved from 1.0 years (95% CI: 0.7–1.2) in 1984–1988 to NR (95% CI: 4.2-NR) in 2014–2018, with an overall median OS of 2.3 years (95% CI: 1.9–2.9). The median GRFS improved from 0.27 years (95% CI: 0.19–0.30) in 1999–2003 to 0.38 years (95% CI: 0.36–0.42) in 2014–2018, with an overall GRFS of 0.38 years (95% CI: 0.36–0.40) (Table 5). The 5-year PFS among the groups showed an improvement over the years: 24% (1984–1988), 25% (1989–1993), 25% (1994–1998), 28% (1999–2003), 33% (2004–2008), 41% (2009–2013), and 48% (2014–2018). Significant improvement was seen since 2009 when compared with the reference group (1984–1988). The 5-year OS increased overall: 25% (1984–1988), 28% (1989–1993), 28% (1994–1998), 28% (1999–2003), 40% (2004–2008), 47% (2009–2013), and 53% (2014–2018). Significant improvement compared with the reference group (1984–1989) was seen since 2004. The 1-year GRFS significantly increased from 12% (1999–2003) to 23% (2009–2013), but no significant improvement was seen in the 2014–2018 group (18%).

Cox proportional hazard models were each established for PFS, OS, and GRFS. Overall, these models confirmed the findings from the Kaplan–Meier analysis for PFS, OS, and GRFS. However, GRFS modeling showed no significant increased risk among the different donor types (i.e., match-related, matched-unrelated, mismatch-related, and mismatch-unrelated), while OS modeling indicated significantly increased risk with haploidentical and mismatched-unrelated donors and PFS modeling showed increased risk in mismatch-unrelated donors only (*p* = 0.022). Donor gender mismatch (M–F and F–M) predicted worse OS outcomes (*p* = 0.049 and *p* = 0.006, respectively), while only F–M mismatch was a factor in PFS outcomes (*p* = 0.003). Myeloablative compared with reduced-intensity conditioning did not influence outcomes in OS and PFS, but marginally predicted worse GFRS outcomes (*p* = 0.052), likely owing to its role on GVHD.

## 4. Discussion

Overall, our data show improved OS, PFS, and GRFS post allo-SCT over decades. We observed an improvement in the rates of several key toxicities including hemorrhagic cystitis, veno-occlusive disease (VOD), and infectious complications. One factor that likely contributed to the decrease in these toxicities was the increased use of RIC regimens, particularly in older patients. The use of high-dose cyclophosphamide in conditioning regimens has also decreased over time, which likely accounts for the reductions in the incidence of hemorrhagic cystitis [24,25]. Several factors likely contributed to lower rates of VOD. For one, the increased use of RIC regimens likely led to lower rates of VOD, as MA regimens have been shown to have higher rates of VOD. The utilization of ursodiol for VOD prophylaxis starting in the early 2000s also likely contributed to lower rates of VOD, as this agent has been shown to decrease the incidence of hepatic aGVHD [26] and hepatic veno-occlusive disease [27,28]. Infectious complications, including bacteremia, viremia, and fungemia, also decreased over the years, which can likely be attributed to the development of more potent infectious disease prophylaxis. Our institution also implemented more sensitive viral testing in the early 2000s, which led to earlier detection and, consequently, prevention of more severe infections. With the increase in peripheral blood stem cells as the preferred source in recent years, faster ANC engraftment may also play a role in the decreased rates of infectious disease [29].

While our study showed a decrease in several key complications over time, it demonstrated an overall increase in the rate of grade II–IV aGVHD, but a relative stability of grade III–IV aGVHD. Several other studies also examined trends in aGVHD over time. In a single-center study, Gooley et al. first compared allo-SCT in 1993–1997 to 2003–2007 and later performed similar comparison between cohorts in 2003–2007 to 2013–2018 [12,16]. The results showed statistically significant reductions in grade III–IV acute GVHD between the 1993–1997 and 2003–2007 groups, but no change between the 2003–2007 and 2013–2018 groups. We observed a significant improvement in NRM over the years. Similar results have been seen in other studies [12,14,15]. As we perform more haploidentical and mismatch-unrelated allo-SCTs, it is important that we continue to optimize management of aGVHD, as rates of aGVHD have been shown to be higher with these sources. Options for steroid-refractory aGVHD have greatly expanded over the years, but there still is no definitive treatment option [30] and it remains an area of active research. One key medication that was recently approved for steroid refractory aGVHD is ruxolitinib, which has been shown to achieve overall response rates of up to 60%. However, additional agents are needed for patients who are either refractory to or are unable to tolerate ruxolitinib [31].

Relapse of primary disease continues to be the biggest challenge with allo-SCT. Our study showed increased risk of relapse of the years after adjusting for covariates. While Penack et al. and Mcdonald et al. showed a decrease in relapse, the improvements were minimal [15,16]. Additionally, previous studies on the role of unrelated donors compared with related donors on relapse have been inconclusive, though hypothetically, the role of the graft-versus-disease effect should be protective against relapse in unrelated donor allo-SCT [32,33]. Our study demonstrated, in comparison with match-related, a reduced risk of relapse in match-unrelated and mismatch-unrelated donor allo-SCTs. However, the general improvements in NRM, OS, and PFS can likely be attributed to improvement in supportive measures, including GVHD and infection prophylaxis, as well as reduced toxicity of conditioning regimens. Moreover, with better treatment, most of the patients recover and continue to live well after weaning off steroids and other immunosuppressive treatments. In concordance with prior studies, both OS and PFS for allo-SCT improved across the years [15,16].

Our study also conducted an analysis using the novel GRFS composite endpoint, useful for understanding the ideal recovery period with a balance between disease cure and ongoing morbidity [20]. The one-year GRFS in recent years (2014–2018) continues to be disappointing for allo-SCT, with approximately one in five patients reaching the one-year mark with ideal recovery. Upon further multivariable analysis, our study showed that donor type did not significantly predict GRFS. A poor response status predicted worse GRFS outcomes, while reduced-intensity conditioning predicted better outcomes.

Overall, in the context of several other studies, our study contributes to foundational knowledge to guide future work in allo-SCT. While aGVHD increased over the years, we saw a decrease in NRM and improvements in both OS and PFS. With GRFS being a composite endpoint balancing both aGVHD and relapse, focus should be on relapse mitigation and development of better treatment regimens for acute GVHD. We are aware of the limitations of our study. The retrospective design could introduce selection bias into our patient cohort. We were limited by the data variables available. Additionally, the increase in aGVHD II–IV could be because of the fact that there was an improvement in documentation in more recent years. Continued efforts to evaluate quality of life following allo-HCT will also be essential to tailor treatment to optimize patient-reported outcomes and minimize morbidity. Both treatment of relapse and potential maintenance therapies following transplant to mitigate relapse are also important areas of research and are the subject of several clinical trials. Studies are needed on these mechanisms and interventions for relapse to bring lasting clinical change following allo-SCTs.

## Figures and Tables

**Table 1:** Patient characteristics based on year of patients undergoing allo-SCT.

	All (n = 1943)		1984–1988 (n = 112)		1989–1993 (n = 107)		1994–1998 (n = 154)		1999–2003 (n = 221)		2004–2008 (n = 252)		2009–2013 (n = 498)		2014–2018 (n = 599)		*p*-Value
	N	%	x̄ /N	SD/%	x̄ /N	SD/%	Mean/N	x̄ /N	x̄ /N	SD/%	x̄ /N	SD/%	x̄ /N	SD/%	Mean/N	SD/%	
Age at SCT, mean, SD	48.1	13.9	31.1	8.5	35.6	9.5	42.2	9.7	44.6	11.7	48.7	12.7	50.5	13.2	54.0	13.1	<0.001
Age at SCT, median, range	50.0	18–76	31.0	18–53	36.0	18–62	43.0	20–65	46.0	21–68	50.0	20–75	53.0	18–73	57.0	18–76	
Donor age, mean, SD	37.4	15.4	31.4	9.6	36.3	10.4	36.4	17.3	40.4	16.5	37.4	19.5	37.8	14.1	37.6	14.7	<0.001
Donor age, median, range	36.0	0–81	31.0	5–61	36.0	8–64	39.0	0–70	42.0	0–78	39.0	0–81	36.0	18–73	33.0	14–79	
Gender, Patients																	0.20
Female	785	40.4	42	37.5	48	44.9	61	39.6	82	37.1	86	34.1	215	43.2	251	41.9	
Male	1158	59.6	70	62.5	59	55.1	93	60.4	139	62.9	166	65.9	283	56.8	348	58.1	
Gender, Donor																	<0.001
Male	1267	65.7	63	56.3	51	47.7	73	49.3	117	53.9	162	65.1	357	71.8	444	74.2	
Female	656	34.0	49	43.8	56	52.3	75	50.7	100	46.1	86	34.5	140	28.2	150	25.1	
Mix of F and M	5	0.3	0	0.0	0	0.0	0	0.0	0	0.0	1	0.4	0	0.0	4	0.7	
Recipient-donor gender																	<0.001
M–M	782	40.7	47	42.0	31	29.0	49	33.1	81	37.3	110	44.4	207	41.6	257	43.3	
M–F	368	19.1	23	20.5	28	26.2	41	27.7	56	25.8	53	21.4	76	15.3	91	15.3	
F–M	485	25.2	16	14.3	20	18.7	24	16.2	36	16.6	52	21.0	150	30.2	187	31.5	
F–F	288	15.0	26	23.2	28	26.2	34	23.0	44	20.3	33	13.3	64	12.9	59	9.9	
Race, patients																	0.13
Caucasian	1830	94.3	111	99.1	105	98.1	143	92.9	212	95.9	240	95.2	465	93.4	554	93.0	
African American	89	4.6	1	0.9	2	1.9	11	7.1	8	3.6	9	3.6	25	5.0	33	5.5	
Others	21	1.1	0	0.0	0	0.0	0	0.0	1	0.5	3	1.2	8	1.6	9	1.5	
Zip code																	0.02
Rural	1231	63.4	74	66.1	68	63.6	99	64.3	139	62.9	178	70.6	307	61.6	366	61.1	
Sub urban	444	22.9	30	26.8	24	22.4	23	14.9	47	21.3	46	18.3	121	24.3	153	25.5	
Urban	268	13.8	8	7.1	15	14.0	32	20.8	35	15.8	28	11.1	70	14.1	80	13.4	
Diagnosis																	<0.001
AA	37	1.9	5	4.5	2	1.9	6	3.9	5	2.3	1	0.4	11	2.2	7	1.2	
ALL	229	11.8	15	13.4	13	12.1	15	9.7	18	8.1	30	11.9	58	11.6	80	13.4	
AML	705	36.3	40	35.7	29	27.1	46	29.9	67	30.3	85	33.7	199	40.0	239	39.9	
MM	55	2.8	1	0.9	6	5.6	5	3.2	16	7.2	11	4.4	5	1.0	11	1.8	
CML	196	10.1	37	33.0	42	39.3	47	30.5	28	12.7	16	6.3	12	2.4	14	2.3	
CLL	85	4.4	0	0.0	0	0.0	4	2.6	9	4.1	28	11.1	28	5.6	16	2.7	
HD	70	3.6	4	3.6	3	2.8	4	2.6	12	5.4	16	6.3	14	2.8	17	2.8	
NHL	275	14.2	7	6.3	7	6.5	8	5.2	47	21.3	45	17.9	87	17.5	74	12.4	
MDS	195	10.0	2	1.8	5	4.7	16	10.4	15	6.8	12	4.8	57	11.4	88	14.7	
MPD	89	4.6	0	0.0	0	0.0	3	1.9	4	1.8	7	2.8	26	5.2	49	8.2	
Others	7	0.4	1	0.9	0	0.0	0	0.0	0	0.0	1	0.4	1	0.2	4	0.7	
KPS																	<0.001
<90	540	30.8	9	81.8	11	34.4	66	43.4	65	30.7	60	24.1	153	30.7	176	29.4	
>=90	1213	69.2	2	18.2	21	65.6	86	56.6	147	69.3	189	75.9	345	69.3	423	70.6	
Tissue																	<0.001
BM	630	32.4	112	100.0	107	100.0	154	100.0	86	38.9	14	5.6	37	7.4	120	20.0	
CB	86	4.4	0	0.0	0	0.0	0	0.0	0	0.0	2	0.8	66	13.3	18	3.0	
PB	1227	63.2	0	0.0	0	0.0	0	0.0	135	61.1	236	93.7	395	79.3	461	77.0	
Donor type																	<0.001
Matched related	908	46.7	107	95.5	82	76.6	102	66.2	147	66.5	138	54.8	164	32.9	168	28.0	
Matched unrelated	756	38.9	0	0.0	15	14.0	37	24.0	57	25.8	85	33.7	240	48.2	322	53.8	
Mismatch related	113	5.8	5	4.5	9	8.4	6	3.9	8	3.6	0	0.0	10	2.0	75	12.5	
Mismatch unrelated	166	8.5	0	0.0	1	0.9	9	5.8	9	4.1	29	11.5	84	16.9	34	5.7	
Conditioning																	<0.001
MA	1068	55.0	112	100.0	107	100.0	154	100.0	185	83.7	113	44.8	126	25.3	271	45.2	
RIC	875	45.0	0	0.0	0	0.0	0	0.0	36	16.3	139	55.2	372	74.7	328	54.8	
Comorbidity index, median, range	2	0–12	NA		NA		NA		NA		1	0–6	3	0–12	2	0–10	<0.001
0–1	422	33.8	NA		NA		NA		NA		78	51.7	120	24.1	224	37.4	
2–3	461	36.9	NA		NA		NA		NA		60	39.7	189	38.0	212	35.4	
4–5	278	22.3	NA		NA		NA		NA		12	7.9	140	28.1	126	21.0	
5+	87	7.0	NA		NA		NA		NA		1	0.7	49	9.8	37	6.2	

Abbreviations: SCT, hematopoietic stem cell transplant; x̄, mean; SD, standard deviation; F, female; MA, myeloablative; RIC, reduced-intensity conditioning; KPS, Karnofsky performance status.

**Table 2:** Engraftment and post-transplant response.

	All (n = 1943)		1984–1988 (n = 112)		1989–1993 (n = 107)		1994–1998 (n = 154)		1999–2003 (n = 221)		2004–2008 (n = 252)		2009–2013 (n = 498)		2014–2018 (n = 599)		*p*-Value
ANC engraftment day, median, range	16	2–120	14	10–31	17	9–42	20	3–120	16	8–28	15	8–23	16	2–45	16	6–43	<0.001
Platelet engraftment day, median, range	19	8–758	16	9–65	23	10–94	31	10–230	25	8–112	19	10–387	17	8–242	18	8–758	<0.001
Post-transplant response																	<0.001
CR	1486	76.5	56	50.0	49	45.8	100	64.9	169	76.5	193	76.6	400	80.3	519	86.6	
Less than CR	164	8.4	20	17.9	5	4.7	8	5.2	12	5.4	26	10.3	40	8.0	53	8.8	
Progression	136	7.0	10	8.9	6	5.6	17	11.0	15	6.8	24	9.5	43	8.6	21	3.5	
Not available	157	8.1	26	23.2	47	43.9	29	18.8	25	11.3	9	3.6	15	3.0	6	1.0	

Abbreviations: ANC, absolute neutrophil count; CR, complete response; VOD, veno-occlusive disease.

**Table 3:** Cumulative Incidence of Acute GVHD and chronic GVHD post-transplant.

Year of Transplant	aGVHD 2–4	aGVHD 3–4		cGVHD E + L	cGVHD E
1999–2003			1999–2003		
Day 100	36 (30–43)	21 (16–26)	Day 180	29 (23–35)	21 (16–26)
Day 180	37 (31–43)	22 (17–27)	Day 365	38 (31–44)	27 (21–33)
2004–2008			2004–2008		
Day 100	27 (33–22)	10 (7–14)	Day 180	22 (17–28)	19 (14–24)
Day 180	31 (36–25)	11 (7–15)	Day 365	40 (34–46)	34 (28–40)
2009–2013			2009–2013		
Day 100	38 (42–33)	11 (8–14)	Day 180	15 (12–18)	14 (11–17)
Day 180	44 (49–40)	13 (10–16)	Day 365	34 (29–38)	31 (27–35)
2014–2018			2014–2018		
Day 100	52 (56–48)	19 (16–22)	Day 180	29 (25–32)	26 (23–30)
Day 180	55 (59–51)	21 (18–24)	Day 365	48 (44–52)	44 (40–48)

Abbreviations: E: extensive, L: limited.

**Table 4:** Relapse modeling.

Univariable Modeling	HR	95% CI		*p*
Year of HCT				
1984–1988	1.00			
1989–1993	0.88	0.48	1.60	0.668
1994–1998	1.32	0.79	2.21	0.296
1999–2003	1.25	0.76	2.04	0.376
2004–2008	1.61	1.02	2.55	0.042
2009–2013	1.57	1.02	2.42	0.042
2014–2018	1.12	0.73	1.74	0.601
Multivariable Modeling				
Year of HCT				
1984–1988				
1989–1993	0.91	0.50	1.63	0.744
1994–1998	1.37	0.79	2.37	0.263
1999–2003	1.37	0.82	2.30	0.228
2004–2008	2.12	1.28	3.52	0.004
2009–2013	2.25	1.35	3.74	0.002
2014–2018	1.86	1.12	3.09	0.016
Age at HCT, 10-year	0.92	0.84	1.00	0.05
recipient_donor_gender				
M–M				
M–F	0.79	0.61	1.04	0.09
F–M	1.40	1.12	1.74	0.003
F–F	1.03	0.77	1.38	0.832
Race, patients				
Caucasian				
non-Caucasian	1.14	0.77	1.66	0.515
>Zip code				
Rural				
Sub urban	0.93	0.75	1.16	0.524
Urban	0.72	0.53	0.97	0.031
Diagnosis				
Lymphoid				
Myeloid	1.10	0.87	1.38	0.429
Others	1.50	1.02	2.22	0.04
Donor type				
Matched-related				
Matched-unrelated	0.73	0.60	0.90	0.004
Mismatch-related	0.86	0.58	1.28	0.47
Mismatch-unrelated	0.64	0.45	0.93	0.019
Conditioning				
MA				
RIC	1.58	1.23	2.03	<0.001
Remission status at transplant				
CR				
PR	1.26	0.95	1.68	0.112
<PR	1.46	1.16	1.84	0.001

**Table 5:** Survival and relapse post-transplant.

Year of Transplant	OS	PFS	Relapse	NRM	GRFS
**1984–1988**					
Median, years	1 (0.7–1.2)	0.8 (0.6–1.2)			
Year 1	50 (40–59)	47 (38–56)	13 (7–19)	40 (49–31)	
Year 3	28 (20–36)	27 (19–35)	21 (14–28)	53 (61–43)	
Year 5	25 (17–33)	24 (17–32)	22 (15–30)	54 (62–44)	
Year 10	21 (14–28)	20 (13–27)	23 (16–31)	58 (67–48)	
**1989–1993**					
Median, years	1 (0.5–1.6)	0.9 (0.4–1.3)			
Year 1	50 (41–59)	49 (39–58)	11 (6–18)	38 (29–47)	
Year 3	35 (26–44)	34 (25–43)	19 (12–27)	46 (36–55)	
Year 5	28 (20–37)	25 (17–34)	21 (14–30)	51 (42–60)	
Year 10	23 (16–32)	20 (13–28)	24 (17–33)	55 (45–64)	
**1994–1998**					
Median, years	0.7 (0.4–1.5)	0.5 (0.3–0.9)			
Year 1	46 (38–54)	42 (34–49)	20 (14–26)	42 (34–49)	
Year 3	35 (28–43)	31 (24–39)	25 (18–32)	47 (39–54)	
Year 5	28 (22–36)	25 (19–32)	28 (21–35)	51 (43–58)	
Year 10	23 (17–30)	21 (15–28)	28 (21–35)	55 (47–63)	
**1999–2003**					
Median, years	0.7 (0.5–1)	0.5 (0.3–0.8)			0.3 (0.2–0.3)
Year 1	44 (37–50)	40 (33–46)	19 (15–25)	46 (39–53)	12 (8–16)
Year 3	31 (25–37)	29 (23–35)	23 (18–29)	55 (48–62)	6 (4–10)
Year 5	28 (23–34)	28 (22–34)	24 (19–30)	57 (50–63)	6 (3–10)
Year 10	26 (20–32)	25 (20–31)	25 (19–31)	58 (51–64)	5 (3–9)
**2004–2008**					
Median, years	2.2 (1.4–3.9)	1 (0.7–1.8)			0.4 (0.4–0.4)
Year 1	61 (54–66)	49 (43–55)	31 (26–37)	21 (16–26)	17 (12–22)
Year 3	48 (41–54)	39 (33–45)	35 (29–41)	27 (22–33)	13 (9–17)
Year 5	40 (34–46)	33 (27–39)	38 (32–44)	31 (26–37)	11 (8–15)
Year 10	34 (28–40)	28 (23–34)	40 (34–46)	34 (28–40)	10 (6–14)
**2009–2013**					
Median, years	3.8 (2.6–5.9)	1.9 (1–2.9)			0.4 (0.4–0.5)
Year 1	65 (61–69)	55 (51–59)	30 (26–34)	15 (12–18)	23 (19–27)
Year 3	52 (48–57)	45 (41–49)	36 (32–40)	19 (15–22)	15 (12–18)
Year 5	47 (42–51)	41 (36–45)	38 (33–42)	22 (19–26)	14 (11–17)
**2014–2018**					
Median, years	NR (4.2-NR)	3.7 (2.3-NR)			0.4 (0.4–0.4)
Year 1	70 (67–74)	63 (59–67)	22 (19–25)	15 (13–18)	18 (15–21)
Year 3	59 (55–63)	52 (47–56)	29 (25–32)	20 (17–23)	11 (9–14)

Abbreviations: OS, overall survival; PFS, progression-free survival; NRM, non-relapse mortality; GRFS, GVHD-free relapse free survival.
